# Vorinostat Mitigates Early Brain Injury Following Subarachnoid Hemorrhage Through Modulation of the C3aR1/TGF‐β/Smad Pathway

**DOI:** 10.1002/cns.71080

**Published:** 2026-08-10

**Authors:** Chonghui Zhang, Yifan Xu, Junshan Wan, Jinpeng Wu, Chao Wang, Yugong Feng

**Affiliations:** ^1^ Department of Neurosurgery The Affiliated Hospital of Qingdao University Qingdao People's Republic of China; ^2^ Medical College Qingdao University Qingdao People's Republic of China

**Keywords:** C3aR1, microglia, subarachnoid hemorrhage, TGF‐β signaling, Vorinostat

## Abstract

**Background:**

Excessive microglial activation after subarachnoid hemorrhage (SAH) contributes to early brain injury (EBI) and poor clinical outcomes. This study explores the role of complement C3a receptor 1 (C3aR1) in microglial activation after SAH and evaluates Vorinostat as a potential therapeutic agent.

**Methods:**

Differentially expressed genes were identified from GEO datasets (GSE36791, GSE73378), followed by WGCNA and machine learning to pinpoint key SAH‐related genes. Molecular docking and molecular dynamics simulation identified Vorinostat as a potential C3aR1‐targeting compound. Functional studies were conducted using C3aR1 knockout mice and BV2 microglial cells. Neurological outcomes were assessed through Garcia score, rotarod, and pole tests. Histological staining and immunofluorescence evaluated neuronal injury and microglial activation. ELISA was used to measure plasma C3aR1 and inflammatory markers in SAH patients and correlate them with 6‐month modified Rankin Scale (mRS) scores.

**Results:**

C3aR1 was identified as a key SAH‐related gene and was significantly upregulated after SAH. C3aR1 deficiency alleviated brain edema, neuronal apoptosis, neuroinflammation, and neurological dysfunction in SAH mice. Transcriptomic and functional analyses demonstrated that these protective effects were mediated, at least in part, through activation of the TGF‐β/Smad signaling pathway. In vitro, C3aR1 knockdown suppressed microglial proliferation, migration, phagocytosis, and inflammatory responses, while reducing oxidative stress in co‐cultured neurons; opposite effects were observed following C3aR1 overexpression. Vorinostat exhibited strong binding affinity to C3aR1, reduced C3aR1 protein expression, activated TGF‐β/Smad signaling, and attenuated EBI after SAH. Clinically, plasma C3aR1 levels were significantly elevated in SAH patients, correlated with inflammatory cytokines and 6‐month modified Rankin Scale scores, and independently predicted poor functional outcomes.

**Conclusion:**

C3aR1 promotes microglial overactivation and neuronal injury after SAH, partly via suppression of TGF‐β signaling. Vorinostat attenuates EBI and is associated with reduced C3aR1 expression. Plasma C3aR1 may serve as a prognostic biomarker in SAH.

## Graphical abstract

C3aR1 mediates microglial activation after SAH in part through the TGF‐β/Smad signaling pathway. Vorinostat alleviates early brain injury after SAH by reducing C3aR1 levels.

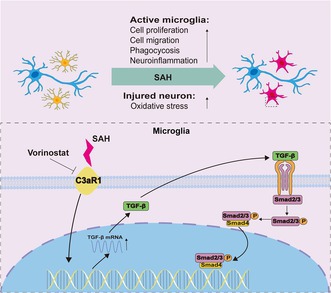

**Abbreviations**
AUC: area under the curve
BBB: blood–brain barrier
C3aR1: complement C3a receptor 1
CCK‐8: cell Counting Kit‐8
DEGs: differentially expressed genes
EBI: early brain injury
ELISA: enzyme‐linked immunosorbent assay
GO‐BP: Gene Ontology Biological Process
IL‐10: interleukin 10
IL‐1Β: interleukin 1β
IL‐4: interleukin 4
IL‐6: interleukin 6
ISPSO: improved variant of particle swarm optimization
qPCR: quantitative real‐time PCR
REOs: relative expression orderings
SAH: subarachnoid hemorrhage
SAHA: suberoylanilide hydroxamic acid
SVM: support vector machine
TOM: topological overlap matrix
TUNEL: terminal deoxynucleotidyl transferase‐mediated dUTP nick‐end labeling
WGCNA: weighted gene co‐expression network analysis

## 1 Introduction

Subarachnoid hemorrhage (SAH), a life‐threatening cerebrovascular event, occurs when cerebral blood vessels rupture and release blood into the subarachnoid cavity [1]. Intracranial aneurysm rupture is the leading cause of SAH. Despite advances in diagnosis and treatment, SAH remains associated with high mortality and poor neurological outcomes [2]. The critical phase of early brain injury (EBI), developing during the initial 3 days post‐hemorrhage, has been identified as the principal factor influencing acute mortality risks and chronic cognitive impairments [3].

As the primary immune defenders within the central nervous system (CNS), microglia become swiftly reactive post‐SAH, secreting inflammatory mediators that subsequently drive blood–brain barrier compromise, programmed cell death in neurons, and oxidative damage [4]. Accumulating evidence suggests that excessive microglial activation exacerbates EBI by modulating the neuroinflammatory microenvironment, though the exact molecular cascades governing this phenomenon require further exploration [5].

Emerging research has identified the complement cascade as a key contributor to neuroinflammation post‐SAH, with particular focus on dysregulated signaling through C3aR1 (complement C3a receptor 1) [6]. C3aR1, a specific receptor for complement component C3a, is highly expressed on the surface of microglia and can enhance their migratory and phagocytic activity upon overactivation [7]. Activation of C3aR1 has been shown to promote the release of pro‐inflammatory cytokines while suppressing anti‐inflammatory responses through downstream pathways such as NF‐κB, thereby exacerbating blood–brain barrier (BBB) disruption and neuronal injury [6, 7]. Clinically, elevated plasma levels of C3a have been detected in SAH patients and correlate positively with disease severity [8, 9]. Despite these findings, the molecular mechanisms by which C3aR1 regulates microglial activation after SAH remain poorly understood.

Vorinostat (suberoylanilide hydroxamic acid, SAHA), a broad‐spectrum histone deacetylase (HDAC) inhibitor approved by the US Food and Drug Administration for the treatment of cutaneous T‐cell lymphoma, has attracted increasing attention for its neuroprotective properties in recent years [10, 11]. In experimental SAH models, SAHA attenuates axonal injury, neurological deficits, neuroinflammation, and microglial pyroptosis [12, 13]. Interestingly, whether the neuroprotective effects of Vorinostat are associated with complement‐mediated neuroinflammatory signaling, particularly the C3aR1 pathway, remains unknown.

Therefore, this study investigated the role of C3aR1 in SAH, evaluated Vorinostat as a potential C3aR1‐targeting therapeutic agent, and assessed the clinical significance of plasma C3aR1.

## 2 Materials and Methods

### 2.1 GEO Data Acquisition and Processing

Microarray datasets comparing SAH patients with healthy individuals were obtained from the GEO database (https://www.ncbi.nlm.nih.gov/geo/). The GSE36791 dataset with 43 SAH and 18 healthy control (HC) samples was used as the training set, while the GSE73378 dataset with 103 SAH and 107 HC samples served as the testing set [14, 15]. Data preprocessing included background correction, normalization, and annotation. Outliers were identified using Bolstad's method [16]. Additionally, we incorporated high‐throughput sequencing data from the GSE167110 dataset, which includes hippocampal tissue from 4 mice with SAH and 3 sham‐operated controls [17].

### 2.2 Differential Analysis and Functional Enrichment Analysis

Differential gene expression analysis was performed using two complementary approaches. First, we applied the LIMMA package to the GSE36791 dataset, following background correction and normalization [18]. Genes with an adjusted *p*‐value (*p*_adj_) < 0.05 and an absolute log_2_ fold change (|log_2_FC|) > 0.585 were considered differentially expressed genes (DEGs). To identify disease‐specific transcriptional dysregulation in a more robust, ranking‐based manner, we additionally employed the RankCompV2 algorithm [19]. This method detects consistent reversal patterns in relative expression orderings (REOs) between gene pairs. Stable REOs observed in healthy controls were defined as background stable REOs, while those exhibiting consistent reversal patterns in SAH patients were termed reversal REOs. Fisher's exact test was used to evaluate the significance of these reversals. Genes showing significant reversal patterns were identified as DEGs by the REO‐based method. The combination of LIMMA and RankCompV2 enhances the reliability of DEG detection by integrating both absolute expression differences and rank‐based regulatory shifts.

For enrichment analyses, we downloaded gene sets from the msigdbr database, including the C5 GO Biological Process collection (c5.go.bp.v2024.1.Hs.symbols.gmt) and canonical pathway gene sets from Reactome and WikiPathways. Functional enrichment and gene set enrichment analyses (GSEA) were performed using the clusterProfiler 4.0 and g:Profiler tools [20]. The enrichment results from g:Profiler were visualized using Cytoscape (v3.10.2).

### 2.3 Weighted Gene Correlation Network Analysis

The WGCNA R package was employed to perform weighted gene co‐expression network analysis, constructing a protein interaction network and identifying functionally relevant modules through systematic computational approaches [21]. Network reliability was enhanced by implementing a scale‐free topology criterion with a threshold *R* ^2^ value of 0.9. To improve correlation accuracy, the primary adjacency matrix was transformed into a topological overlap measure (TOM), effectively filtering stochastic associations. Module detection was carried out using a soft‐threshold power (*β*) of 12, a minimum module size of 20, and a merging threshold (cut height) of 0.15, with dynamic tree cutting implemented via the “cutreeDynamic” function. The resulting modules were visualized using Cytoscape.

Genes within significant modules were subjected to Gene Ontology Biological Process (GO‐BP) enrichment analysis. Subsequently, genes from key modules were intersected with previously identified differentially expressed genes (DEGs), and overlapping genes were considered as high‐confidence candidates for further investigation.

### 2.4 Feature Engineering Based on ISPSO

To identify robust diagnostic features, an improved variant of particle swarm optimization (ISPSO)‐based feature selection strategy was integrated with a support vector machine (SVM) classifier [22]. The model was trained for 100 iterations, and features consistently selected in high‐performing models (AUC > 0.9 in both training and validation sets) were retained for downstream analysis. Gene expression data from GSE36791 were randomly divided into training and validation sets (7:3). SVM models with different kernel functions and box constraint values were optimized using fivefold cross‐validation. The final model was evaluated in the training set, validation set, and an independent test cohort (GSE73378) using AUC as the primary performance metric.

### 2.5 Immune Infiltration Analysis

Immune cell infiltration was analyzed using CIBERSORTx (https://cibersortx.stanford.edu/), which deconvolutes the cellular composition of complex tissues based on gene expression data. Comparative evaluation of immune cell distribution between SAH patients and HCs was conducted using Wilcoxon rank‐sum testing. Spearman's rank correlation coefficients were calculated to quantify relationships between central gene expression patterns and immune cell population densities.

### 2.6 Identification of Key Genes

To facilitate subsequent experimental validation, we analyzed the mouse dataset GSE167110 using the DESeq2 package [23]. DEGs were determined using the threshold of an adjusted *p*‐value (*p*_adj_) < 0.05 and absolute log_2_ fold change (|log_2_FC|) > 0.585. We then intersected the upregulated and downregulated DEGs with the core genes previously identified through ML approaches. This overlap ensured that the selected candidate genes demonstrated consistent and stable expression patterns across both datasets.

### 2.7 Animals

C3aR1 knockout (C3aR1^−/−^) male mice (Strain S‐KO‐16129, Cyagen Biosciences Inc., Suzhou, China) and age‐matched wild‐type C57BL/6 male mice (Beijing Vital River Laboratory Animal Technology Co. Ltd., Beijing, China) were used in this study. All mice were housed under standardized conditions, including a 12‐h light–dark cycle, free access to food and water, relative humidity maintained at 20%–50%, and temperature controlled between 21°C and 25°C.

### 2.8 Anesthesia and Surgery

The experimental protocol for intravascular perforations followed established methodologies as previously outlined. Animals received anesthesia via intraperitoneal injection of 1% pentobarbital sodium solution, after which careful isolation of the right internal carotid artery (ICA) from adjacent tissues was performed under surgical microscopy. A precisely calibrated monofilament nylon suture (0.16 ± 0.01 mm gauge; Beijing Cinontech Co. Ltd., China) was subsequently advanced through the ICA lumen to create controlled perforation at the vascular bifurcation of the middle cerebral and anterior cerebral arteries. Core body temperature regulation was maintained within the range of 37°C ± 0.5°C throughout experimental procedures using automated thermal control systems.

### 2.9 Drug Administration

Vorinostat (Sigma‐Aldrich, St. Louis, MO, USA) was prepared in a solvent mixture containing 10% dimethyl sulfoxide (DMSO) and delivered via intraperitoneal injection 24 h before surgery [24]. For nasal administration, 10 μL of SB‐505124 (360 μM in PBS) was administered intranasally using a cannula (0.2 mm inner diameter) attached to a 10 μL Hamilton microsyringe, with an infusion speed of 12 μL/min. Control groups received equivalent volumes of PBS.

### 2.10 Brain Water Content

Twenty‐four hours following SAH, mouse brains were harvested, and the wet weight of the tissue was immediately recorded. The samples were then dried in an oven at 105°C for 24 h, after which the dry weight was measured. The percentage of water content was calculated using the following formula: ([wet weight − dry weight]/wet weight) × 100%.

### 2.11 Behavioral Analysis

Neurological performance was assessed in a blinded manner using the modified Garcia scoring test, the rotarod test, and the pole test. The modified Garcia test was scored on a scale of 3–18, with higher scores indicating better neurobehavioral outcomes [25]. For the rotarod test, the rotating speed gradually increased from 0 to 30 rpm [26]. The time elapsed until the mice fell off was recorded. In the pole test, the duration needed to perform a 180° rotation was measured as the turning time, while the overall time taken to reach the base of the pole was recorded as the total descent time. If the turn time exceeded 30 s, both turn time and total time were recorded as 30 s [27].

### 2.12 HE and Nissl Staining

HE and Nissl staining were performed on paraffin sections. HE staining involves hematoxylin for nuclei, eosin for cytoplasm, followed by dehydration, clearing, and mounting. Nissl staining uses toluidine blue to highlight Nissl bodies in neural tissue, also with dehydration, clearing, and mounting.

### 2.13 Terminal Deoxynucleotidyl Transferase‐Mediated dUTP Nick‐End Labeling Assay

Apoptosis in brain tissue was assessed using a one‐step terminal deoxynucleotidyl transferase‐mediated dUTP nick‐end labeling (TUNEL) apoptosis in situ detection kit (Keygen, BioTECH, China). The procedure involved fixing and permeabilizing frozen tissue sections, followed by incubation with TdT reaction buffer at 37°C in the dark for 30–60 min. Subsequently, the sections were treated with Streptavidin‐FITC‐labeled solution at 37°C for 1 h. Following thorough rinsing, cellular nuclei were contrasted with DAPI prior to fluorescence microscopic analysis.

### 2.14 Quantitative Real‐Time PCR (qPCR)

Total RNA isolation was conducted with Trizol reagent, followed by cDNA synthesis using the Advantage RT for PCR Kit (Shandong Sparkjade Biotechnology Co. Ltd., China). qRT‐PCR analysis was then conducted with the iQTM SYBR Green Supermix (Yeasen Biotech Co. Ltd., China). Relative gene expression quantification was calculated via the $2^{-\Delta \Delta C_{T}}$ method, with primer sequences detailed in Table S1.

### 2.15 Bulk RNA‐Seq

Twenty‐four hours post‐operation, the cortex was harvested for further analysis (WT group, *n* = 3; KO group, *n* = 3). Total mRNA extraction was conducted using the KAPA Stranded mRNA‐Seq Kit. Subsequently, library preparation was performed after confirming the RNA purity and quantifying its concentration. Following successful quality assessment of the library, sequencing was executed on the Illumina NovaSeq 6000 platform. Differential expression analysis was carried out using DESeq2 [28]. Genes with a *Q* value < 0.05 and fold change > 2 or < 0.5 were designated as significantly DEGs. Additionally, transcriptome sequencing and analysis services were provided by Shanghai Genefund Biotech Co. Ltd.

### 2.16 Western Blot

Cellular and tissue proteins were isolated through RIPA buffer treatment (Solarbio, China). Protein concentrations were measured using a BCA assay kit (Meilun, China). Equivalent protein amounts underwent electrophoretic separation on 10% SDS‐PAGE gels before being electrotransferred onto PVDF membranes (Thermo Fisher Scientific). Membranes were initially treated with protein‐free blocking buffer for 10 min, then exposed overnight at 4°C to primary antibodies against C3aR1 (MBS7125171, MyBioSource, USA), TGF‐β1 (26155‐1‐AP, Proteintech, China), p‐Smad2/3 (80427‐2‐RR, Proteintech, China), Smad2/3 (EPR19557‐4, abcam, UK), and GAPDH (60004‐1‐Ig, Proteintech, China). Following this, the membranes were treated with HRP‐linked secondary antibodies and incubated at ambient temperature for 50 min. Protein bands were visualized using ECL reagents and detected with a chemiluminescent imaging system (Millipore, USA).

### 2.17 Immunostaining and Image Analysis

Tissue sections were prepared following transcardial perfusion and fixation. The sections were washed three times with PBS, blocked with PBS containing 0.3% Triton X‐100 and 5% normal goat serum for 2 h, and incubated overnight at 4°C with anti‐Iba1 (Oasisbiofarm, OB‐PGP049, 1:500, China) diluted in the blocking solution. After washing with PBS, incubate with the secondary antibody at room temperature for 90 min. Image the brain sections using a Leica confocal microscope and evaluate using ImageJ. The Sholl analysis of Iba1 was performed using the Neuroanatomy plugin (https://imagej.net/update‐sites/neuroanatomy/).

### 2.18 Cell Culture, Treatment and Transfection

The BV2 murine microglia cell line (Xiamen Immocell Biotechnology Co. Ltd.) was maintained in growth medium at 37°C with 5% CO_2_ atmosphere. The culture medium consisted of high‐glucose DMEM (Dulbecco's Modified Eagle Medium, Gibco, USA) formulated with 10% fetal bovine serum (FBS, Gibco, USA), supplemented with penicillin (100 U/mL) and streptomycin (100 μg/mL). For establishing the SAH in vitro model, BV2 cells were exposed to OxyHb (10 μM concentration, Sigma‐Aldrich, USA) dissolved in complete culture medium. Parallel cultures without Hb treatment were designated as control groups.

C3aR1‐knockdown cellular models were generated via lentivirus‐mediated transduction. Following transduction, cells underwent selection in complete culture medium containing 3 μg/mL puromycin (MedChemExpress, Shanghai, China). For C3aR1 overexpression studies, plasmid vectors were utilized to generate gain‐of‐function cellular models. Plasmid DNA or empty vector controls were transfected into BV2 cells using Lipofectamine 3000 reagent (Invitrogen, USA). Transfected cells were collected 24 h post‐transfection for subsequent experimental analyses. All lentiviral constructs and plasmid vectors were custom‐designed and manufactured by OBiO Technology (Shanghai, China), with corresponding sequence details provided in Table S2.

### 2.19 Cell Viability Assay

Cell viability was determined with the CCK‐8 detection kit (Beyotime, China) following standardized protocols. BV2 microglial cells were plated in 96‐well culture plates and allowed to adhere overnight under standard culture conditions. After initial culture, different concentrations of OxyHb (0–80 μM) were administered to the cells for 24‐h treatment. Following this incubation period, 10 μL of CCK‐8 solution was introduced to each well, with subsequent 2‐h incubation before absorbance measurement.

### 2.20 Cell Proliferation Assay

The EdU incorporation method was employed to evaluate cellular proliferation dynamics. EdU‐labeled cells were visualized using the BeyoClick EdU Cell Proliferation Detection Kit (containing Alexa Fluor 647, Beyotime, China) according to the manufacturer's protocol. The proliferation rate was calculated as the percentage of EdU‐positive cells.

### 2.21 Transwell Migration Assay

A Transwell assay was used to evaluate BV2 cell migration. Transfected cells were serum‐starved and placed in upper chambers, with 10% FBS in lower chambers as a chemoattractant. After 24 h at 37°C, migrated cells were fixed, stained, and counted. Migration capacity was calculated based on average counts from three random fields.

### 2.22 Phagocytosis Assay

BV2 cells were cultured in a 6‐well plate at a density of 400,000 cells per well and transfected with lentivirus or plasmid following the previously outlined procedure. Latex beads (L4655, Sigma, USA) were introduced into the plate and incubated at 37°C for 30 min. After collection, the BV2 cells were resuspended in PBS and subsequently analyzed using flow cytometry.

### 2.23 Cell Co‐Culture System and ROS Assay

BV2 and HT22 cells were indirectly co‐cultured using a Transwell system (0.4 μm pore size; Corning, USA). Pretreated BV2 cells were seeded in the upper chamber, whereas HT22 cells were cultured in the lower chamber. After 24 h of co‐culture, intracellular ROS levels in HT22 cells were detected using 10 μM DCFH‐DA (Beyotime, S0033S). Fluorescence intensity was analyzed by microscopy and quantified using ImageJ software.

### 2.24 Molecular Docking

Potential therapeutic compounds targeting C3aR1 were screened using the Drug Signature Database (DSigDB). The top‐ranked candidate compounds were subjected to molecular docking analysis using AutoDock Vina 1.5.7 to evaluate their binding affinities with C3aR1. Drug structures were obtained from the PubChem database, and the C3aR1 protein structure was retrieved from the Protein Data Bank (PDB). The binding conformations with the lowest binding energies were visualized using PyMOL 3.1.

### 2.25 Molecular Dynamics Simulation

The study uses GROMACS 2024‐2 for high‐throughput MD simulations with four stages: preparation, minimization, equilibration, and production. Protein topology is generated with amber14sb force field and TIP3P water model, while ligand parameters are created with sobtop. The system undergoes energy minimization and equilibration at 300 K and 1 bar before running 50 ns production simulations with a 2 fs time step. Bonds are constrained during equilibration, and LINCS resets bond lengths in the final stage [29].

### 2.26 Participants

Patients with aneurysmal subarachnoid hemorrhage (aSAH) caused by intracranial aneurysm rupture were consecutively recruited between March 2023 and October 2024. The diagnosis was confirmed by brain computed tomography (CT) and vascular imaging. Eligible patients were aged ≥ 18 years and underwent plasma collection within 24 h after SAH onset. Age‐matched healthy volunteers without a history of brain injury, neurological disorders, or bleeding disorders were enrolled as controls. Functional outcomes were assessed using the modified Rankin Scale (mRS) at 6 months after SAH and categorized as favorable (mRS 0–2) or unfavorable (mRS 3–6). Plasma samples were centrifuged at 2500 × *g* for 10 min at 4°C and stored at −80°C until analysis.

### 2.27 Enzyme‐Linked Immunosorbent Assay Detection

To explore the impact of C3aR1 on inflammatory molecules, enzyme‐linked immunosorbent assay (ELISA) was employed to measure the levels of C3aR1, human interleukin 6 (IL‐6), human interleukin 1β (IL‐1β), human interleukin 4 (IL‐4), and human interleukin 10 (IL‐10) in the plasma of participants. The ELISA kits were supplied by Jiangsu Meimian Industrial Co. Ltd. An initial experiment was performed to identify the optimal dilution ratio, and all subsequent steps were carried out in strict accordance with the manufacturer's protocol.

### 2.28 Statistical Analysis

RNA‐seq data were subjected to statistical analysis using R Studio (v 4.3.0). Statistical analyses were conducted with the GraphPad Prism 10 software. Data are expressed as mean ± standard error of the mean (SEM), and significance levels are denoted as follows: *p < 0.05, **p < 0.01, ***p < 0.001, and ****p < 0.0001. For pairwise comparisons, unpaired Student's *t*‐tests were employed. Multiple comparisons were analyzed using one‐way or two‐way ANOVA, followed by Tukey's post hoc test. Spearman correlation analysis was performed to evaluate the relationships between C3aR1 and IL‐6, IL‐1β, IL‐4, IL‐10, as well as the mRS score. The area under the receiver operating characteristic (ROC) curve and its corresponding 95% confidence interval (CI) were calculated. Sensitivity and specificity values for each independent risk factor were determined across various critical thresholds. Multivariate logistic regression analysis was performed to identify independent predictors of unfavorable functional outcome after SAH. Odds ratios (ORs) with 95% confidence intervals (CIs) were calculated. *p* < 0.05 was considered statistically significant.

## 3 Results

### 3.1 Differential Expression and Functional Enrichment Analysis

**FIGURE 1:**
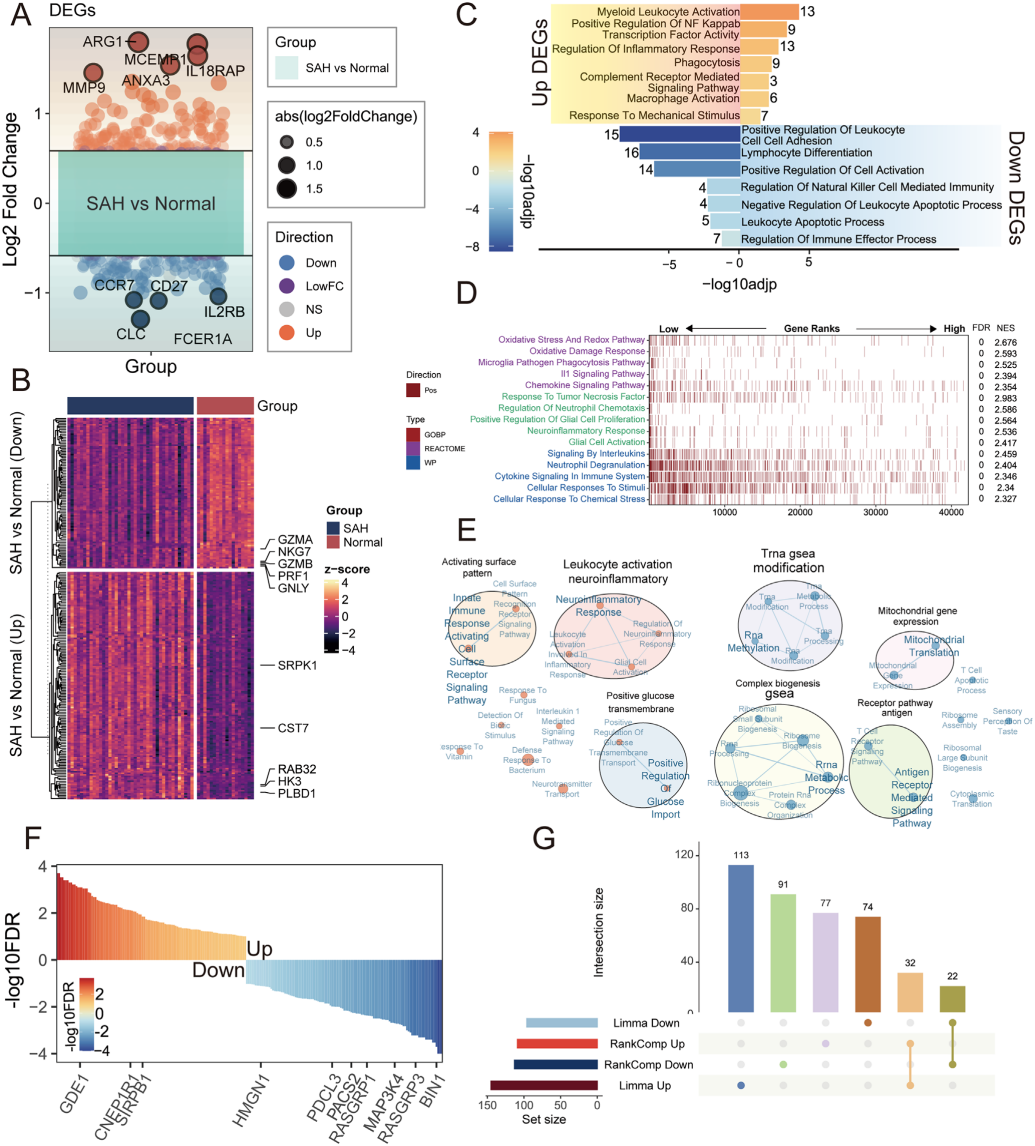
Differential gene expression and functional enrichment analyses in peripheral blood from SAH patients based on the GSE36791 dataset. (A, B) Volcano plot and heatmap illustrating DEGs identified through the LIMMA package analysis, highlighting significant upregulated (red) and downregulated (blue) genes. (C) Functional enrichment analysis of the DEGs. (D) Gene set enrichment analysis (GSEA). (E) Cytoscape visualization integrating GSEA results. (F, G) RankCompV2 algorithm analysis identifying robustly DEGs between SAH patients and controls.

Differential expression analysis of the GSE36791 dataset identified a series of significantly dysregulated genes in SAH patients (Figure 1A,B). Functional enrichment analysis revealed that upregulated genes were mainly associated with inflammatory and immune‐related processes, including myeloid leukocyte activation, NF‐κB signaling, and regulation of inflammatory responses, whereas downregulated genes were enriched in pathways related to leukocyte activity and lymphocyte maturation (Figure 1C). GSEA further demonstrated significant enrichment of oxidative stress, microglial phagocytosis, IL‐1 signaling, and chemokine signaling pathways in SAH (Figure 1D,E), highlighting the involvement of immune activation and oxidative stress in SAH pathogenesis. In addition, RankCompV2 analysis identified 32 upregulated and 22 downregulated genes between SAH patients and healthy controls (Figure 1F,G).

### 3.2 Identification of Diagnostic Genes for SAH Based on WGCNA and Machine Learning

**FIGURE 2:**
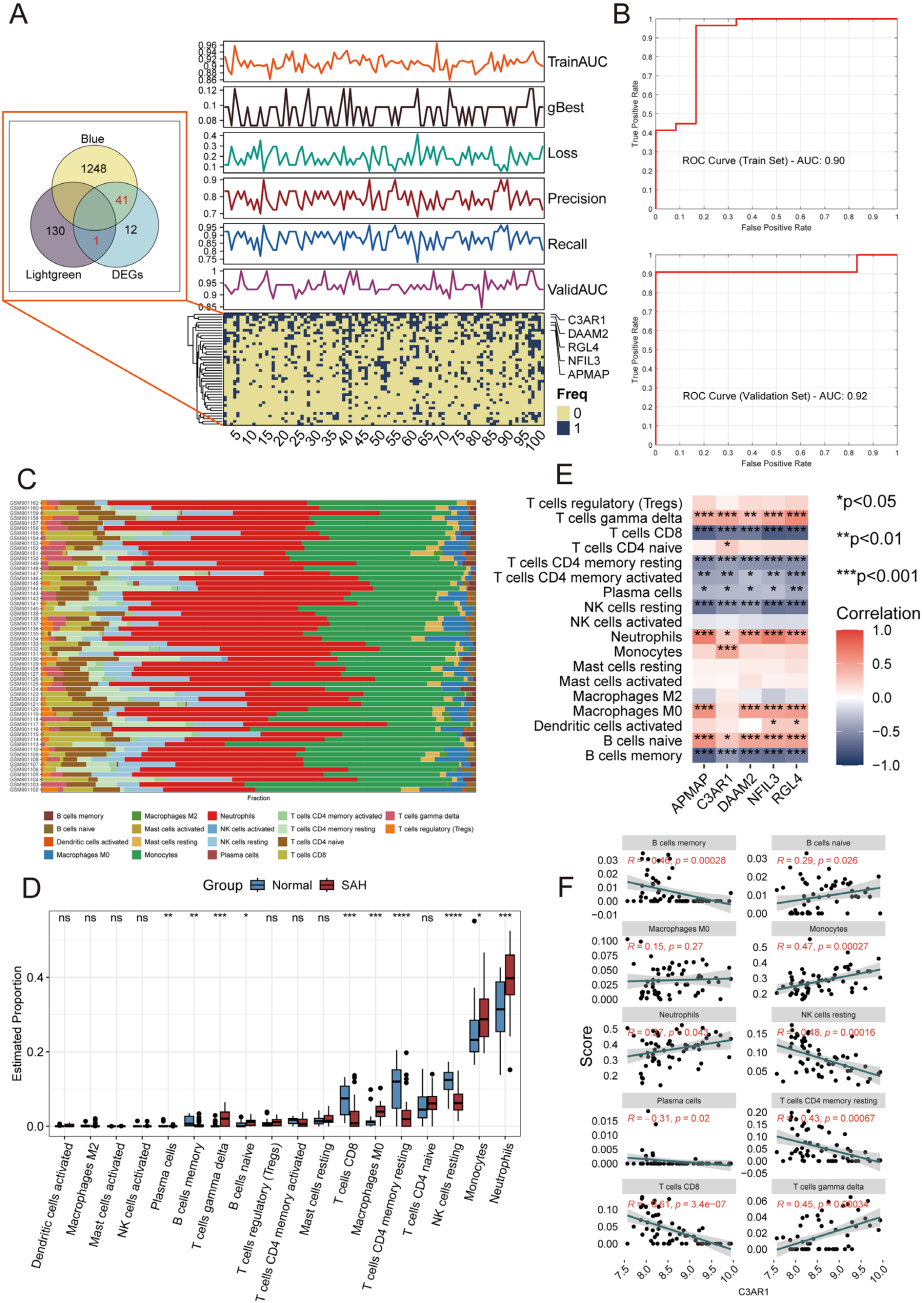
Machine learning‐based identification of key diagnostic genes and immune infiltration landscape in SAH using the GSE36791 dataset. (A) Integration of DEGs with WGCNA key modules (blue and light green) followed by feature selection using the ISPSO algorithm combined with an SVM model. (B) Performance of the final SVM classifier constructed using five selected key genes (C3AR1, DAAM2, RGL4, NFIL3, and APMAP). (C, D) Immune cell infiltration analysis by CIBERSORTx in peripheral blood samples from SAH patients and healthy controls. (E, F) Correlation heatmap depicting associations between expression levels of the five diagnostic genes and proportions of immune cell populations.

WGCNA identified several gene co‐expression modules significantly associated with SAH, among which the black, blue, darkorange, lightgreen, and pink modules showed the strongest correlations with disease status (all *p* < 0.01; Figure S1A–D). Functional enrichment analysis indicated that these modules were primarily involved in immune regulation, protein degradation, tissue repair, and immune defense processes (Figure S1E). By integrating DEGs with key WGCNA modules, ISPSO‐SVM analysis identified five hub genes (C3AR1, DAAM2, RGL4, NFIL3, and APMAP) as core diagnostic features. The resulting classifier demonstrated robust performance, with AUC values of 0.90 and 0.92 in the training and test sets, respectively (Figure 2A,B). Immune infiltration analysis further revealed increased proportions of neutrophils, monocytes, and M0 macrophages, accompanied by reduced CD4+ memory resting T cells, CD8+ T cells, and resting NK cells in SAH patients (Figure 2C,D), indicating a pronounced immune activation state.

### 3.3 C3aR1 Emerges as a Central Hub Gene in SAH Pathogenesis and Its Immunoregulatory Features

To explore potential immune interactions, we performed correlational analyses on the GSE36791 dataset, demonstrating significant positive correlations between C3AR1 expression levels and the abundance of neutrophils, monocytes, and M0 macrophage subtypes (Figure 2E,F). These data suggest C3AR1's potential role in shaping the immune microenvironment dynamics during SAH progression via cross talk with diverse immune cell populations.

**FIGURE 3:**
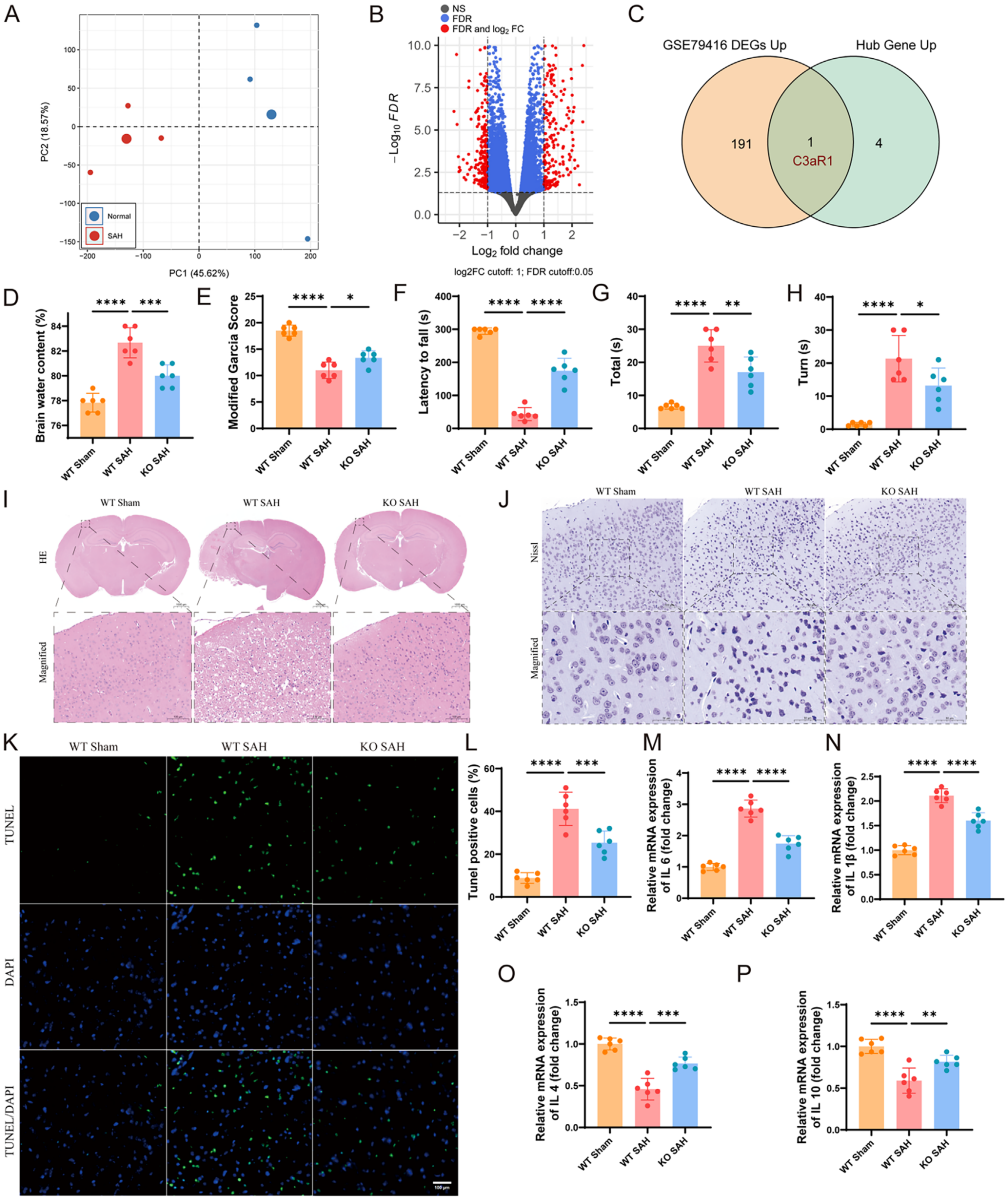
Effects of C3aR1 knockout on neurological function, brain injury, apoptosis and neuroinflammation in SAH mice. (A) Principal component analysis (PCA) of SAH and control samples in GSE167110. (B) Volcano plot of differentially expressed genes (DEGs). (C) Venn diagram showing the overlap between upregulated DEGs and hub genes identified from key modules. (D–H) Brain water content, Modified Garcia scores, rotarod performance, and pole test results in different groups. (I, J) Representative HE and Nissl staining images of brain sections. (K, L) Representative TUNEL staining images and quantification of TUNEL‐positive cells in cortical tissues. (M–P) Relative mRNA expression levels of IL‐6, IL‐1β, IL‐4, and IL‐10 in cortical tissues. *n* = 6 per group. *p < 0.05, **p < 0.01, ***p < 0.001, ****p < 0.0001.

Differential gene expression analysis was conducted on the murine SAH model dataset GSE79416. Principal component analysis (PCA) (Figure 3A) demonstrated clear separation between SAH and normal control samples. The volcano plot (Figure 3B) identified significantly differentially expressed genes, with thresholds set at |log_2_FC| > 1 and FDR < 0.05; red dots represent significantly upregulated genes meeting both criteria. Intersection analysis between the upregulated DEGs and hub genes from key modules (Figure 3C) revealed a single overlapping gene, C3aR1, suggesting its potential central role in SAH pathogenesis.

### 3.4 C3aR1 Knockout Attenuates Early Brain Injury in SAH Mice

C3aR1 knockout was confirmed by western blot analysis, which showed nearly undetectable cortical C3aR1 expression in KO mice (Figure S2). Compared with WT mice, C3aR1 deficiency significantly reduced brain edema and improved neurological function after SAH, as evidenced by higher Modified Garcia scores, longer rotarod latency, and shorter total and turn times in the pole test (Figure 3D–H). Histological analyses further demonstrated that C3aR1 knockout alleviated neuronal degeneration and preserved Nissl body integrity following SAH (Figure 3I,J). Consistently, the number of TUNEL‐positive cells was significantly reduced in KO mice compared with WT mice (Figure 3K,L). In addition, C3aR1 deficiency decreased the mRNA expression of pro‐inflammatory cytokines (IL‐6 and IL‐1β) while increasing the expression of anti‐inflammatory cytokines (IL‐4 and IL‐10), indicating an attenuation of SAH‐induced neuroinflammation (Figure 3M–P).

### 3.5 C3aR1 Knockout May Inhibit Microglia Overactivation in SAH Mice by Activating TGF‐β Signaling Pathway

**FIGURE 4:**
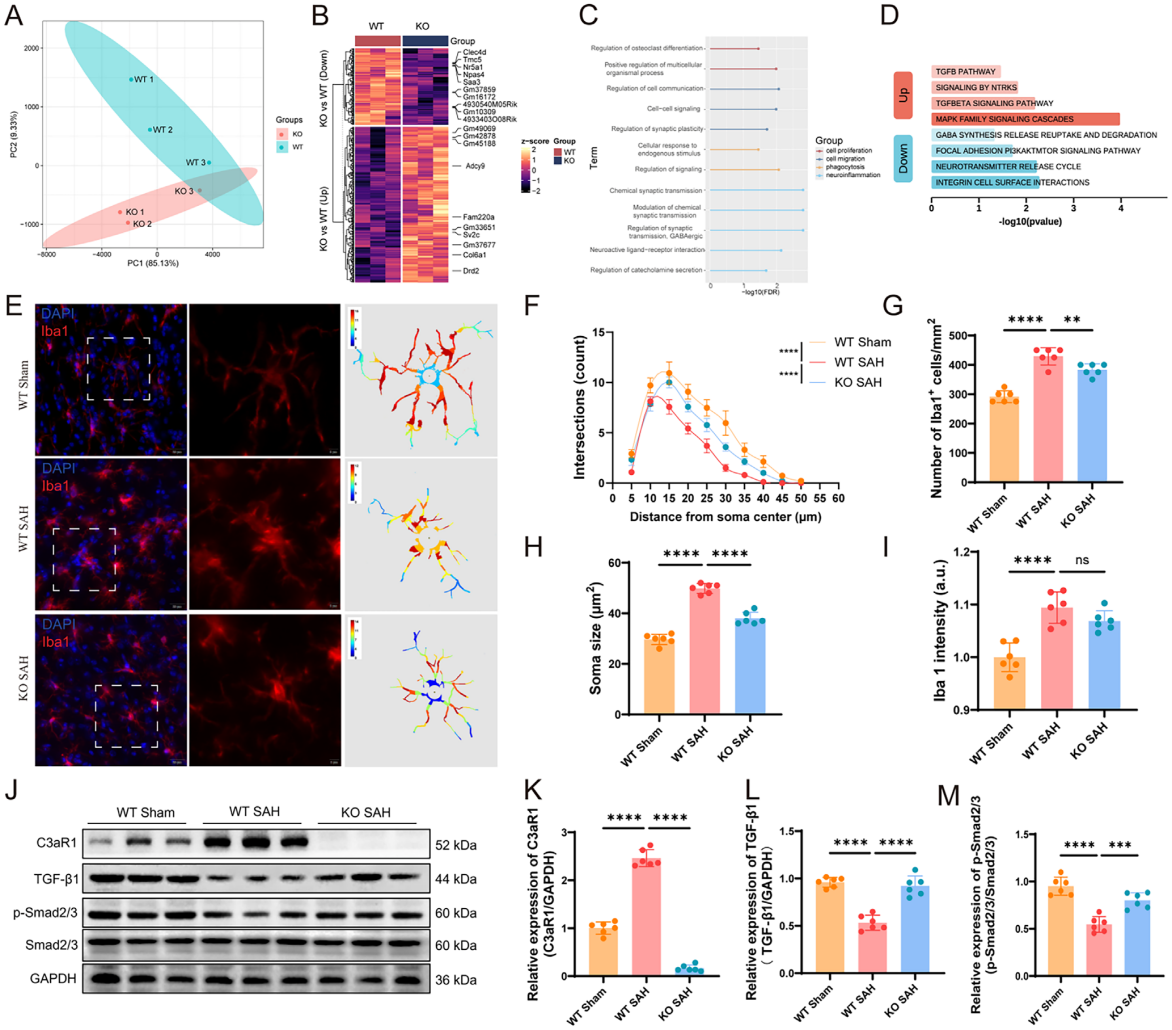
Bulk RNA‐seq analysis revealed the activation of microglia and alterations in the TGF‐β signaling pathway in C3aR1 knockout mice. (A) Principal component analysis (PCA) of cortical transcriptomes from WT and C3aR1 KO mice after SAH. (B) Heat map of differentially expressed genes between WT and KO mice. (C, D) GO enrichment analysis of differentially expressed genes. (E) Representative immunofluorescence images of microglia (Iba1, red; DAPI, blue). (F) Sholl analysis of microglial morphology. (G–I) Quantification of microglial number, soma size, and fluorescence intensity. (J) Representative western blot images. (K–M) Quantification of C3aR1, TGF‐β1, and p‐Smad2/3 protein expression. *n* = 6 per group. **p < 0.01, ***p < 0.001, ****p < 0.0001.

To investigate the mechanisms underlying the protective effects of C3aR1 deficiency after SAH, bulk RNA‐seq was performed on cortical tissues from WT and KO mice. Transcriptomic analysis revealed distinct expression profiles between the two groups (Figure 4A,B). Functional enrichment analysis indicated that differentially expressed genes were mainly associated with cell activation, neuroinflammation, and the TGF‐β signaling pathway (Figure 4C,D and Figure S3).

Immunofluorescence analysis showed that SAH induced marked microglial activation, characterized by reduced branching complexity, increased cell number, enlarged cell volume, and enhanced Iba1 fluorescence intensity, whereas these changes were significantly attenuated in C3aR1 KO mice (Figure 4E–I). Consistent with these findings, western blot analysis demonstrated that TGF‐β1 and p‐Smad2/3 protein expression were significantly decreased after SAH but were partially restored by C3aR1 deficiency (Figure 4J–M), suggesting activation of the TGF‐β/Smad pathway.

Furthermore, treatment with the TGF‐β receptor inhibitor SB‐505124 abolished the neuroprotective effects of C3aR1 knockout, resulting in worsened neurological outcomes and reduced Smad2/3 phosphorylation (Figure S4). These findings indicate that activation of the TGF‐β/Smad pathway contributes to the protective effects mediated by C3aR1 deficiency after SAH.

### 3.6 In Vitro, C3aR1 Knockdown Suppressed Microglial Proliferation, Migration, and Phagocytosis, and Decreased ROS Levels in Neurons

**FIGURE 5:**
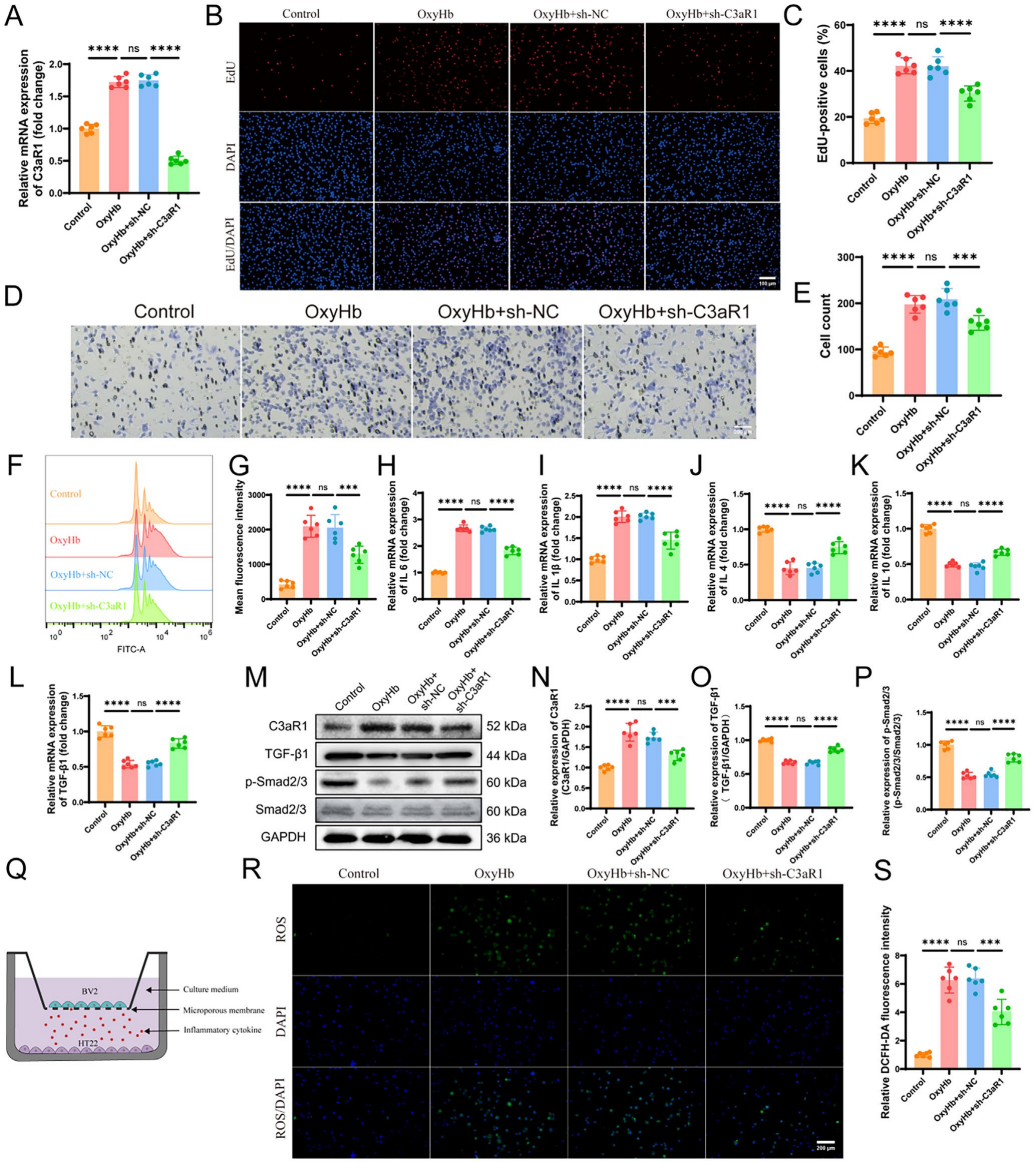
Effect of knockdown of C3aR1 on the functions of BV2 and HT22 cells. (A) Relative C3aR1 mRNA expression in BV2 cells under different treatment conditions. (B, C) Representative EdU staining images and quantification of BV2 cell proliferation. (D, E) Representative Transwell images and quantification of BV2 cell migration. (F, G) Flow cytometric analysis and quantification of latex bead phagocytosis by BV2 cells. (H–L) Relative mRNA expression levels of IL‐6, IL‐1β, IL‐4, IL‐10, and TGF‐β1 in BV2 cells. (M) Representative western blot images. (N–P) Quantification of C3aR1, TGF‐β1, and p‐Smad2/3 protein expression. (Q) Schematic diagram of the BV2–HT22 co‐culture system. (R, S) Representative ROS staining images and quantification of intracellular ROS levels in HT22 cells. *n* = 6 per group. **p < 0.01, ***p < 0.001, ****p < 0.0001.

To investigate the role of C3aR1 in microglial activation, an OxyHb‐induced BV2 cell model was established, and 10 μM OxyHb was selected for subsequent experiments based on CCK‐8 analysis (Figure S5). Lentiviral transfection efficiently reduced C3aR1 expression (Figure 5A). Functional assays showed that C3aR1 knockdown significantly inhibited OxyHb‐induced proliferation, migration, and phagocytic activity of BV2 cells (Figure 5B–G). Moreover, C3aR1 knockdown reduced the mRNA expression of pro‐inflammatory cytokines (IL‐6 and IL‐1β) while increasing the expression of anti‐inflammatory cytokines (IL‐4 and IL‐10), indicating an attenuation of OxyHb‐induced microglial inflammatory responses (Figure 5H–K). OxyHb significantly increased iNOS expression and decreased Arg1 expression at both the mRNA and protein levels. C3aR1 knockdown reversed these effects, indicating that inhibition of C3aR1 suppresses OxyHb‐induced microglial M1 polarization (Figure S6). The above results confirmed the role of C3aR1 in promoting microglia neuroinflammation. C3aR1 knockdown increased TGF‐β1 expression and partially reversed the OxyHb‐induced reduction in TGF‐β1 and p‐Smad2/3 protein levels, indicating activation of the TGF‐β/Smad signaling pathway (Figure 5L–P). Furthermore, in the BV2–HT22 co‐culture system, C3aR1 knockdown significantly attenuated the increase in neuronal ROS levels induced by OxyHb‐stimulated microglia (Figure 5Q–S).

### 3.7 In Vitro, C3aR1 Overexpression Promoted Microglial Proliferation, Migration, and Phagocytosis, While Increasing ROS Levels in Neurons

**FIGURE 6:**
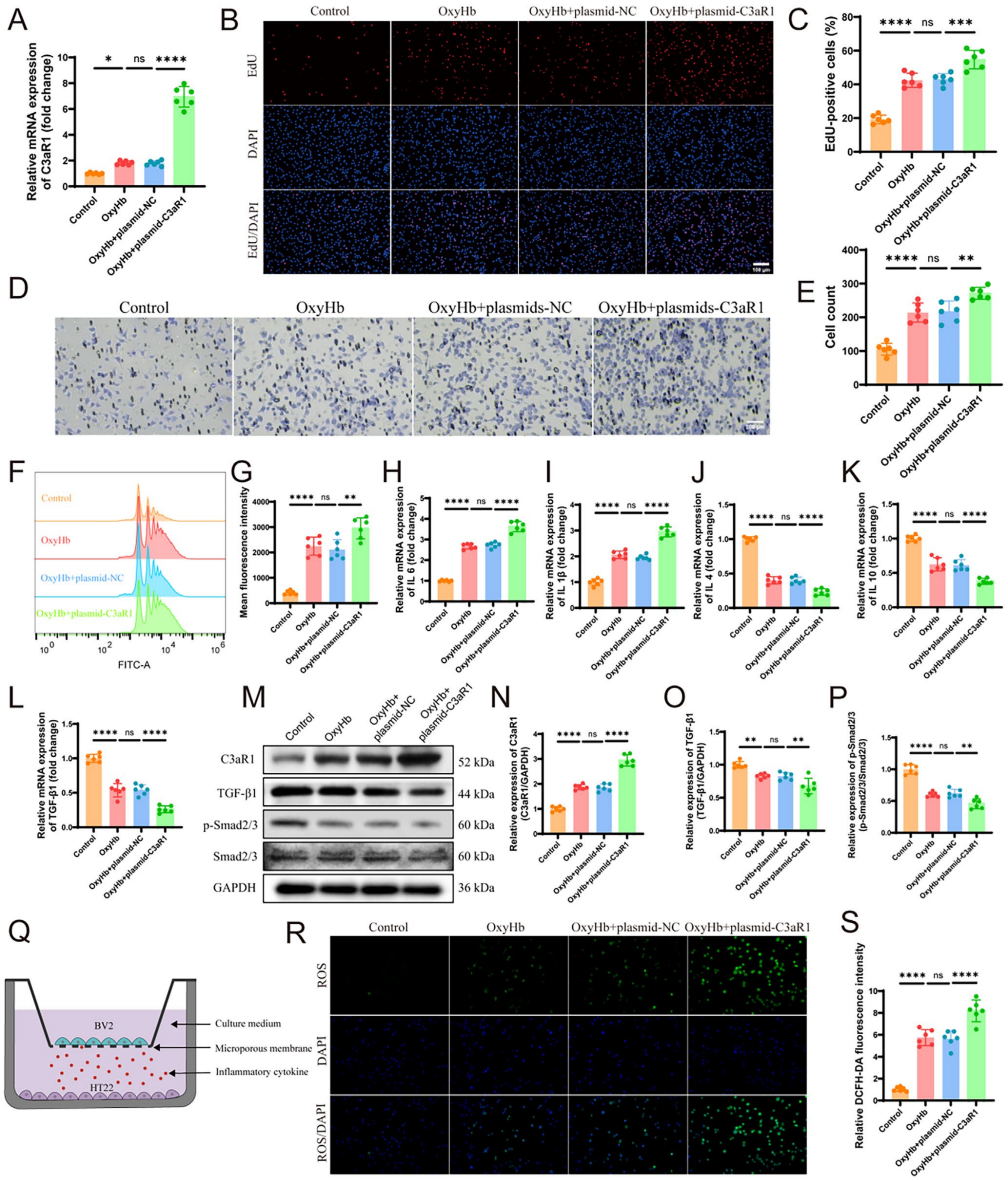
Effect of overexpression of C3aR1 on the functions of BV2 and HT22 cells. (A) Relative C3aR1 mRNA expression in BV2 cells under different treatment conditions. (B, C) Representative EdU staining images and quantification of BV2 cell proliferation. (D, E) Representative Transwell images and quantification of BV2 cell migration. (F, G) Flow cytometric analysis and quantification of latex bead phagocytosis by BV2 cells. (H–L) Relative mRNA expression levels of IL‐6, IL‐1β, IL‐4, IL‐10, and TGF‐β1 in BV2 cells. (M) Representative western blot images. (N–P) Quantification of C3aR1, TGF‐β1, and p‐Smad2/3 protein expression. (Q) Schematic diagram of the BV2–HT22 co‐culture system. (R, S) Representative ROS staining images and quantification of intracellular ROS levels in HT22 cells. *n* = 6 per group. *p < 0.05, **p < 0.01, ***p < 0.001, ****p < 0.0001.

Plasmid transfection efficiently increased C3aR1 expression in BV2 cells (Figure 6A). Functional assays demonstrated that C3aR1 overexpression significantly enhanced OxyHb‐induced proliferation, migration, and phagocytic activity of microglia (Figure 6B–G). In addition, C3aR1 overexpression increased the expression of pro‐inflammatory cytokines (IL‐6 and IL‐1β) while reducing the expression of anti‐inflammatory cytokines (IL‐4 and IL‐10), indicating an exacerbation of microglial inflammatory responses (Figure 6H–K). Consistently, C3aR1 overexpression further promoted M1 polarization, as evidenced by increased iNOS and decreased Arg1 expression (Figure S7).

Mechanistically, C3aR1 overexpression further suppressed TGF‐β1 expression and aggravated the OxyHb‐induced reduction in TGF‐β1 and p‐Smad2/3 protein levels, indicating inhibition of the TGF‐β/Smad signaling pathway (Figure 6 L–P). Furthermore, in the BV2–HT22 co‐culture system, C3aR1‐overexpressing microglia significantly increased ROS accumulation in HT22 neurons (Figure 6Q–S).

### 3.8 Vorinostat Is a Potential Therapeutic Agent Targeting C3aR1

**FIGURE 7:**
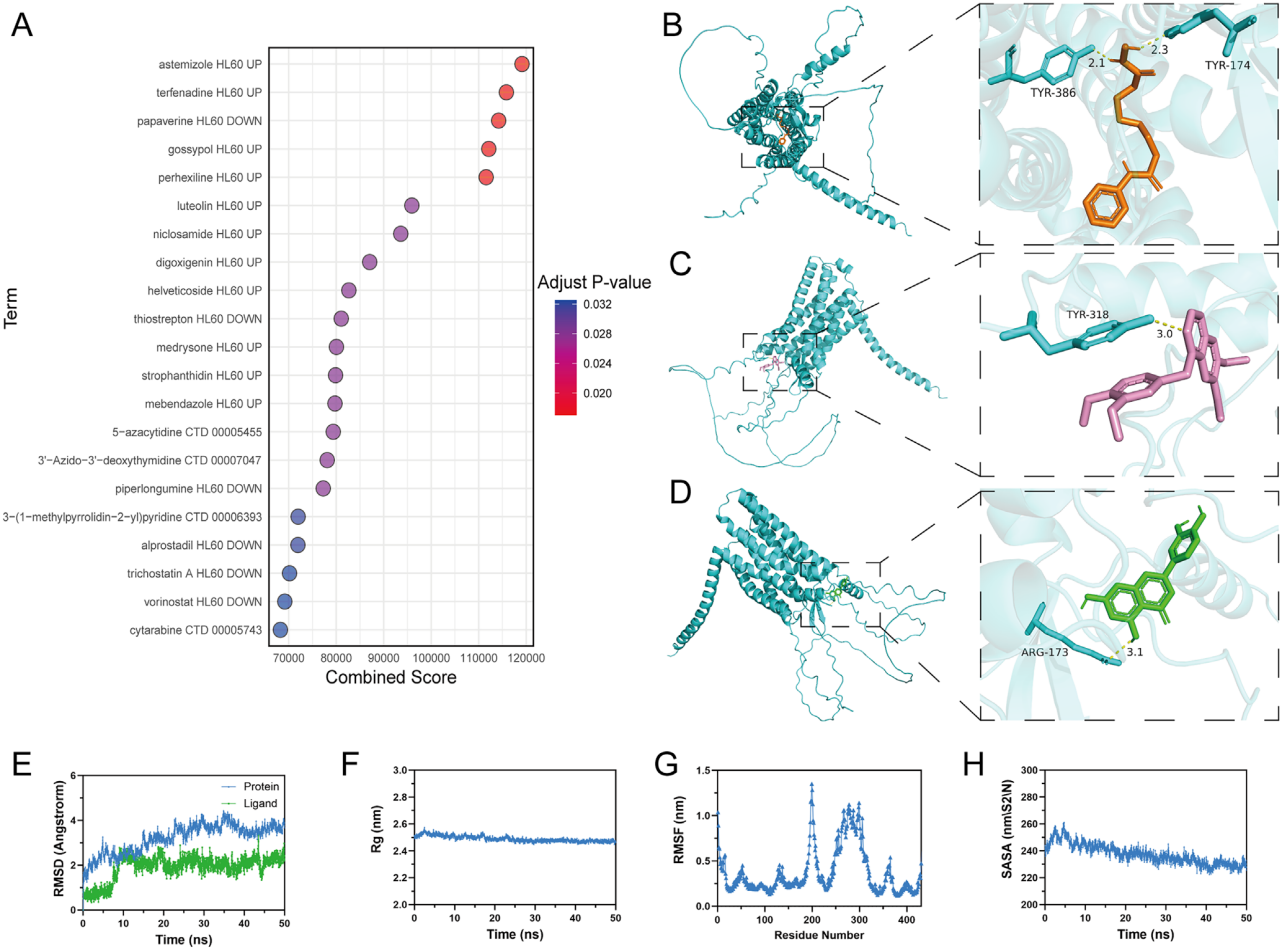
Molecular docking and kinetic simulation were used to screen available small molecules targeting C3aR1. (A) The Top 20 small molecules for C3aR1 predicted by the DSigDB database. (B–D) The docking results of C3aR1 with vorinostat (orange), papaverine (pink), and luteolin (green). (E) Evolution of RMSD (root mean square deviation) during 50 ns molecular dynamics simulations of vorinostat (ligand) and C3aR1 (pocket). (F) Evolution of Rg (radius of gyrate) during 50 ns molecular dynamics simulations of C3aR1. G RMSF (root mean square fluctuation) of C3aR1. (H) SASA (accessible solvent surface area) of C3aR1 during 50 ns molecular dynamics simulations.

DSigDB analysis identified multiple potential compounds targeting C3aR1, with the top 20 candidates shown in Figure 7A. Molecular docking revealed that vorinostat, papaverine, and luteolin exhibited the strongest binding affinities toward C3aR1, with vorinostat showing the lowest binding energy (−8.7 kcal/mol) (Table S3). The binding conformations of these compounds with C3aR1 are presented in Figure 7B–D.

Given its highest binding affinity, vorinostat was selected for molecular dynamics simulation. Analysis of RMSD, Rg, RMSF, and SASA demonstrated that the vorinostat‐C3aR1 complex remained stable throughout the simulation, supporting a favorable and stable interaction between vorinostat and C3aR1 (Figure 7E–H).

### 3.9 Vorinostat Alleviates Microglial Activation and Early Brain Injury in SAH Mice by Downregulating C3aR1 Expression and Activating the TGF‐β Signaling Pathway

**FIGURE 8:**
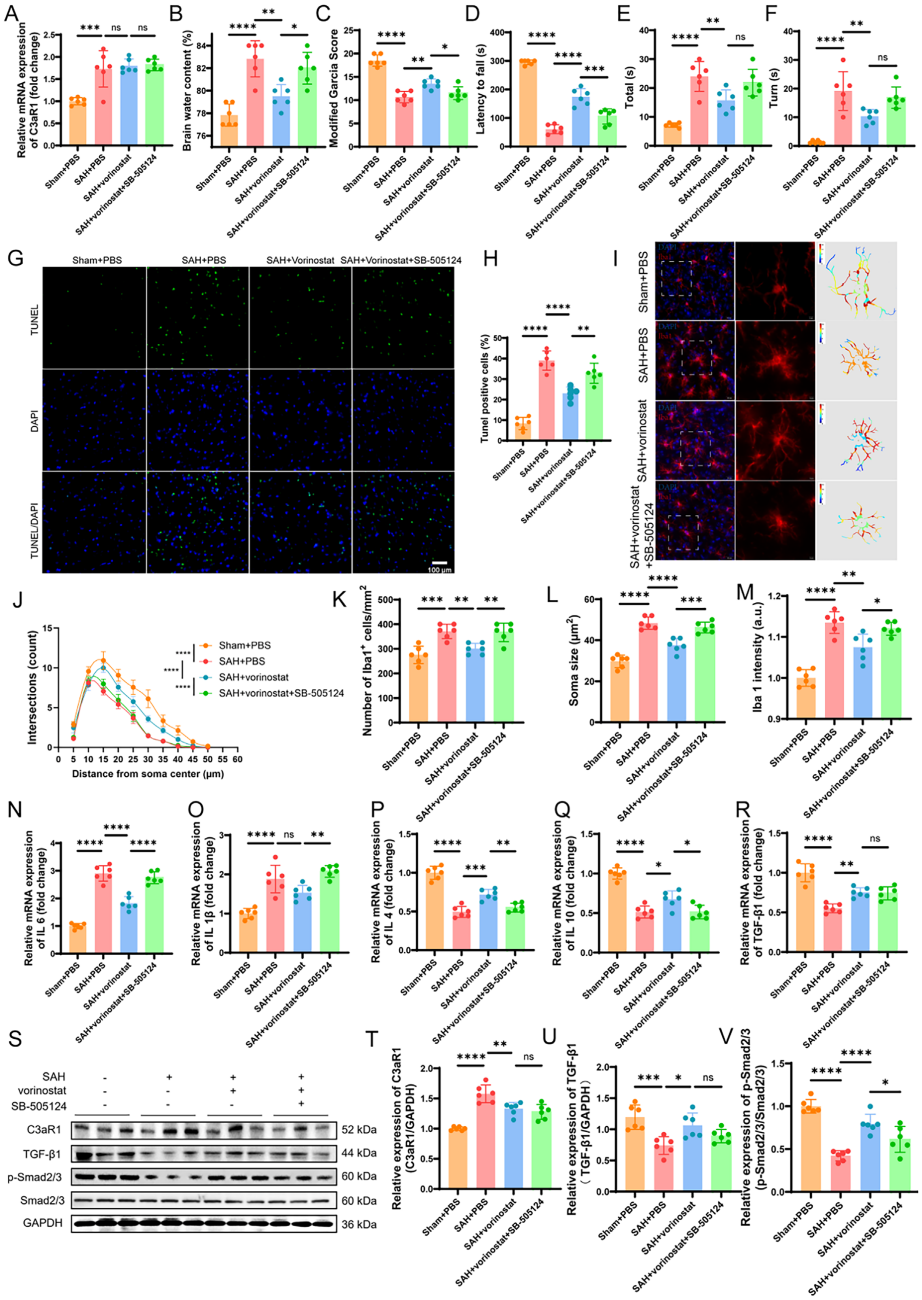
Effects of Vorinostat on neurological function, apoptosis, microglia activation and TGF‐β signaling pathway in SAH mice. (A) Relative C3aR1 mRNA expression in cortical tissues. (B) Brain water content. (C) Modified Garcia scores. (D) Rotarod performance. (E, F) Total time and turn time in the pole test. G, H Representative TUNEL staining images and quantification of TUNEL‐positive cells. (I) Representative immunofluorescence images of microglia (Iba1). (J) Sholl analysis of microglial morphology. (K–M) Quantification of microglial number, soma size, and fluorescence intensity. (N–R) Relative mRNA expression levels of IL‐6, IL‐1β, IL‐4, IL‐10, and TGF‐β1 in cortical tissues. S Representative western blot images. (T–V) Quantification of C3aR1, TGF‐β1, and p‐Smad2/3 protein expression. *n* = 6 per group. *p < 0.05, **p < 0.01, ***p < 0.001, ****p < 0.0001.

Previous analyses identified Vorinostat as a potential C3aR1‐targeting compound. We therefore investigated its effects on neurological function, apoptosis, and microglial activation after SAH, while examining the involvement of TGF‐β signaling using the selective TGF‐β receptor inhibitor SB‐505124. Dose–response experiments showed that Vorinostat at 50 and 100 mg/kg significantly reduced cortical C3aR1 protein expression, whereas lower doses had no obvious effect. Accordingly, 50 mg/kg was selected for subsequent experiments. Vorinostat did not significantly affect body weight recovery after SAH (Figure S8). Notably, Vorinostat treatment reduced C3aR1 protein expression without altering C3aR1 mRNA levels, suggesting that its effect may be mediated through direct interaction with C3aR1 rather than transcriptional regulation (Figure 8A).

Vorinostat significantly reduced brain edema and improved neurological function after SAH, as evidenced by improved modified Garcia scores, prolonged rotarod latency, and improved pole test performance (Figure 8B–F). These beneficial effects were largely abolished by SB‐505124, although inhibition of TGF‐β signaling did not significantly affect the reduction in total time observed in the pole test. Consistently, TUNEL staining demonstrated that Vorinostat markedly reduced neuronal apoptosis after SAH, whereas SB‐505124 reversed this protective effect (Figure 8G,H).

Immunofluorescence analysis revealed that SAH induced marked microglial activation, characterized by increased microglial number, reduced branching complexity, and enlarged cell bodies (Figure 8I–M). Vorinostat partially reversed these alterations, whereas SB‐505124 largely abolished its effects. Similarly, Vorinostat decreased the expression of pro‐inflammatory cytokines IL‐6 and IL‐1β while increasing the expression of anti‐inflammatory cytokines IL‐4 and IL‐10; whereas SB‐505124 shifted the inflammatory profile toward that observed in SAH mice (Figure 8N–Q).

Western blot analysis further demonstrated that Vorinostat reduced C3aR1 protein expression and increased TGF‐β1 expression at both the mRNA and protein levels (Figure 8R–U). This increase in TGF‐β1 expression was not affected by SB‐505124 treatment. In contrast, SB‐505124 effectively suppressed Smad2/3 phosphorylation without altering TGF‐β1 expression (Figure 8V), consistent with its role as a TGF‐β type I receptor inhibitor. Collectively, these findings indicate that Vorinostat alleviates EBI after SAH by suppressing C3aR1‐mediated microglial activation and promoting TGF‐β/Smad signaling.

### 3.10 Expression of C3aR1, IL 6, IL 1β, IL 4, IL 10 in Plasma and the Correlation Between C3aR1 and IL 6, IL 1β, IL 4, IL 10, mRS Scores

**FIGURE 9:**
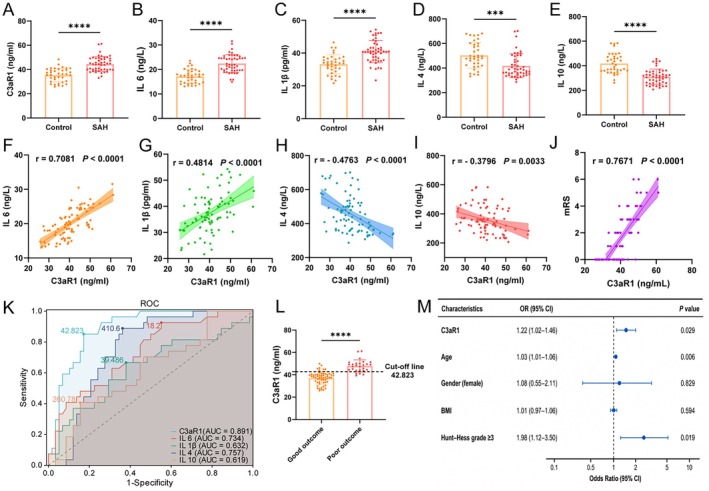
Expression of C3aR1, IL 6, IL 1β, IL 4, IL 10 in plasma and the correlation between C3aR1 and IL 6, IL 1β, IL 4, IL 10, mRS scores. (A–E) The level of C3aR1, IL 6, IL 1β, IL 4, IL 10 in plasma were detected by ELISA in control and SAH patients. (F–J) The relationship between C3aR1 and IL 6, IL 1β, IL 4, IL 10 and mRS scores. (K) ROC showed that plasma C3aR1 levels after SAH had a better diagnostic value for outcome at 6‐month follow‐up. (L) The cutoff value of C3aR1 was 42.823 ng/mL according to the ROC curve. (M) Multivariate logistic regression analysis identifying independent predictors of poor functional outcome after SAH. The model was adjusted for age, sex, body mass index, hypertension, diabetes mellitus, smoking status, alcohol consumption, and Hunt–Hess grade. ***p < 0.001, ****p < 0.0001.

To investigate the clinical significance of C3aR1 in SAH, plasma samples from 48 patients with SAH and 37 healthy controls were analyzed by ELISA. Compared with controls, SAH patients exhibited significantly higher levels of C3aR1, IL‐6, and IL‐1β, accompanied by lower levels of IL‐4 and IL‐10 (Figure 9A–E). Correlation analysis demonstrated that plasma C3aR1 levels were positively associated with IL‐6, IL‐1β, and 6‐month mRS scores, whereas negative correlations were observed with IL‐4 and IL‐10 (Figure 9F–J).

ROC analysis was performed to evaluate the prognostic value of C3aR1 and inflammatory cytokines. Among all biomarkers examined, C3aR1 showed the highest predictive performance, with an AUC of 0.891, sensitivity of 85.19%, and specificity of 82.76% (Figure 9K and Table S4). The optimal cut‐off value for plasma C3aR1 was 42.823 ng/mL (Figure 9L). Baseline characteristic analysis revealed that patients with poor outcomes were older and had higher Hunt–Hess grades than those with favorable outcomes, whereas no significant differences were observed in sex, BMI, hypertension, diabetes mellitus, smoking status, or alcohol consumption (Table S5). Multivariate logistic regression analysis demonstrated that elevated plasma C3aR1 levels were independently associated with poor functional outcome after SAH (OR = 1.22, 95% CI: 1.02–1.46, *p* = 0.029; Figure 9M).

## 4 Discussion

In the present study, integrated bioinformatics and machine learning analyses identified C3aR1 as a key regulator in SAH. Experimental studies demonstrated that C3aR1 was upregulated after SAH, and its deficiency alleviated EBI, neuronal apoptosis, neuroinflammation, and microglial activation. Mechanistically, these protective effects were associated with activation of the TGF‐β/Smad signaling pathway. In vitro, C3aR1 promoted microglial proliferation, migration, phagocytosis, and pro‐inflammatory responses, leading to increased neuronal oxidative stress. In addition, Vorinostat, identified through molecular docking and molecular dynamics analyses, reproduced the protective effects of C3aR1 deficiency in experimental SAH. Clinically, elevated plasma C3aR1 levels were associated with poor neurological outcomes. Collectively, these findings suggest that C3aR1 contributes to SAH‐induced brain injury by promoting microglial activation and suppressing TGF‐β/Smad signaling.

To improve the reliability of biomarker identification, we integrated multiple bioinformatics and machine learning approaches, including LIMMA, RankCompV2, and ISPSO‐SVM analysis. This strategy enabled robust identification of disease‐associated genes and yielded a predictive model with good performance across independent datasets, supporting the reliability of C3aR1 as a candidate biomarker in SAH [18]. It is well established that secondary brain injury following SAH occurs in two distinct phases: EBI and delayed cerebral ischemia (DCI) [30]. Among them, EBI—occurring within the first 72 h post‐hemorrhage—has been recognized as a major contributor to mortality and neurological deficits [31]. Accumulating evidence indicates that activation and polarization of microglia play a central role in EBI progression [32]. As the principal immune effector cells in the central nervous system, microglia contribute to neuronal apoptosis, synaptic dysfunction, and inflammatory responses following SAH [33]. The C3aR1‐mediated complement signaling pathway is known to regulate microglial activity in a time‐dependent manner: short‐term exposure to C3a enhances phagocytosis, while prolonged stimulation may suppress immune function [34]. In our study, knockdown of C3aR1 in vitro significantly inhibited microglial proliferation, migration, and phagocytic capacity, highlighting its importance in microglial immune activation. In vivo experiments further confirmed that C3aR1 deletion attenuated EBI in SAH mice, as evidenced by reduced neuronal apoptosis and decreased expression of pro‐inflammatory cytokines. Collectively, these results suggest that C3aR1 contributes to EBI through microglial activation, supporting its potential as a neuroprotective therapeutic target.

C3aR1 is predominantly expressed in microglia within the CNS and modulates their function via multiple signaling pathways [35]. Previous studies have demonstrated that C3aR1 regulates microglial homeostasis through HIF‐1α signaling and promotes microglial activation and phagocytosis under pathological conditions [35, 36]. Our findings identify the C3aR1/TGF‐β axis as a potential mechanism linking microglial activation to neuroinflammation following SAH. Notably, beyond its effects on microglial function, C3aR1 also plays a key role in cell‐to‐cell communication between neurons, astrocytes, and microglia [37]. Literature reports suggest that amyloid‐β (Aβ) induces C3 expression in astrocytes, which under chronic stimulation suppresses microglial Aβ phagocytosis via a C3aR1‐dependent mechanism, thereby exacerbating Aβ pathology [34]. Consistent with these findings, C3aR1 knockdown attenuated the increase in neuronal ROS levels induced by OxyHb‐stimulated microglia, further supporting a role for C3aR1 in microglia‐mediated neuronal injury after SAH.

Given the pathogenic role of C3aR1, identifying potential pharmacological inhibitors is of considerable therapeutic interest. Through silico drug screening and molecular docking, we found that SAHA exhibits a high predicted binding affinity to C3aR1, suggesting that modulation of C3aR1 signaling may contribute to its neuroprotective effects. In the context of SAH, SAHA has been reported to attenuate axonal injury and neurological deficits by promoting acetylation of HSP70 and degradation of TDP‐43 [38]. Moreover, SAHA alleviates neuroinflammation and microglial pyroptosis in SAH, further supporting its potential for CNS protection [38, 39]. Our in vivo experiments confirmed that SAHA ameliorates neurological dysfunction in SAH mice by modulating the C3aR1/TGF‐β signaling pathway, reducing neuronal apoptosis and suppressing microglial overactivation. These findings not only expand our understanding of SAHA's pharmacological action but also support its potential as a targeted therapeutic agent in SAH.

Despite these promising findings, several limitations should be acknowledged. First, only male mice were included in this study; therefore, future studies involving both sexes are needed to validate the role of C3aR1 and the therapeutic effects of Vorinostat after SAH. Second, although pharmacological inhibition of TGF‐β signaling abolished the neuroprotective effects of C3aR1 deficiency, the present study did not investigate potential physical interactions between C3aR1 and TGF‐β pathway components. Future studies using co‐immunoprecipitation, proximity ligation assays, or other molecular approaches are warranted to further clarify the mechanistic relationship between these pathways. Third, plasma C3aR1 was measured at a single time point, limiting assessment of its temporal dynamics and prognostic value. Lastly, although C3aR1 is predominantly expressed in microglia in the CNS, its cellular distribution after SAH was not systematically investigated in the present study. Future studies employing co‐localization analyses and single‐cell transcriptomics may provide a more comprehensive characterization of C3aR1 localization in the injured brain and further elucidate the origin, cellular distribution, and biological significance of circulating and membrane‐bound C3aR1 after SAH.

## 5 Conclusion

Experimental studies demonstrated that C3aR1 deficiency alleviated EBI and suppressed microglial activation, at least in part, through the TGF‐β/Smad signaling pathway. Furthermore, Vorinostat, a potential C3aR1‐targeting compound, exhibited significant neuroprotective effects in experimental SAH. These findings provide new insights into the mechanisms of neuroinflammation after SAH and highlight C3aR1 as a promising therapeutic target.

## Author Contributions

Chonghui Zhang was responsible for designing the research framework and drafting the primary content of the manuscript. Chao Wang and Yifan Xu conducted the data preprocessing and performed the analysis. The preparation of all tables was completed by Junshan Wan and Jinpeng Wu. Yugong Feng contributed to reviewing and revising the manuscript. All authors have reviewed the final version of the manuscript and given their approval for its submission.

## Funding

The authors have nothing to report.

## Ethics Statement

This study was approved by the Ethics Committee of the Affiliated Hospital of Qingdao University (approval number: QYFYWZLL30310) and all participants provided written informed consent. All animal experiments were approved by the Animal Care and Use Committee of Qingdao University (20250401C576J12020260501171).

## Consent

The authors have nothing to report.

## Conflicts of Interest

The authors declare no conflicts of interest.

## Supporting information


**Table S1:** Primers used in qRT‐PCR.
**Table S2:** shRNA and plasmids targeting C3aR1 used in this study.
**Table S3:** Docking results of C3aR1 with small molecules.
**Table S4:** ROC information for each variable at its optimal cutoff value.
**Table S5:** Baseline characteristics of patients with SAH.
**Figure S1:** Weighted gene co‐expression network analysis (WGCNA) of the GSE36791 dataset identifies gene modules associated with SAH.
**Figure S2:** Validation of C3aR1 expression levels in KO mice.
**Figure S3:** Bulk RNA‐seq revealed pathways enriched for differentially expressed genes between the two groups of mice (KO vs. WT).
**Figure S4:** SB‐505124 abolished the neuroprotective effects of C3aR1 deficiency after SAH.
**Figure S5:** The cell viability of BV2 cells treated with different concentrations of OxyHb.
**Figure S6:** C3aR1 knockdown attenuates OxyHb‐induced M1 polarization in microglia.
**Figure S7:** C3aR1 overexpression exacerbates OxyHb‐induced M1 polarization in microglia.
**Figure S8:** Dose optimization of Vorinostat and its effects on body weight after SAH.

## Data Availability

The data that support the findings of this study are available from the corresponding author upon reasonable request.
