# Supplementary material for: Vorinostat Mitigates Early Brain Injury Following Subarachnoid Hemorrhage Through Modulation of the C3aR1/TGF‐β/Smad Pathway
Source: CNS Neurosci Ther. 2026 Aug 10;32(8):e71080. doi: 10.1002/cns.71080 (PMC13455684; doi:10.1002/cns.71080)
Supplement: Supplementary file 1 [file CNS-32-e71080-s001.docx]

**Table S1. Primers used in qRT-PCR.**

| **Primer** | **Sequence 5′-3′** |
| --- | --- |
| C3ar1 (Forward) | ATACACTGAACGCTGACGCT |
| C3ar1 (Reverse) | GAAAAACGGCACCAGGAAGC |
| TGF-β1 (Forward) | AGCTGCGCTTGCAGAGATTA |
| TGF-β1 (Reverse) | AGCCCTGTATTCCGTCTCCT |
| IL 6 (Forward) | ACAACGATGATGCACTTGCAGA |
| IL 6 (Reverse) | TGGTACTCCAGAAGACCAGAGGA |
| IL 1β (Forward) | ACAAGGAGAACCAAGCAACGAC |
| IL 1β (Reverse) | TTGCTTGGGATCCACACTCTCC |
| IL 4 (Forward) | TCGGCATTTTGAACGAGGTCAC |
| IL 4 (Reverse) | AGACGAGCTCACTCTCTGTGG |
| IL 10 (Forward) | GAGGCGCTGTCATCGATTTCTC |
| IL 10 (Reverse) | AGACACCTTGGTCTTGGAGCTTA |
| GAPDH (Forward) | AGCCCAAGATGCCCTTCAGT |
| GAPDH (Reverse) | CCGTGTTCCTACCCCCAATG |

**Table S2. shRNA and plasmids targeting C3aR1 used in this study.**

|  | Sequence 5′-3′ |
| --- | --- |
| shRNA-C3aR1(F) | CTACCAGAAAGCAATTCTA |
| shRNA-C3aR1(R) | CTACCAGAAAGCAATTCTA |
| shRNA-NC(F) | CCTAAGGTTAAGTCGCCCTCG |
| shRNA-NC(R) | CCTAAGGTTAAGTCGCCCTCG |
| Plasmids-C3aR1(F) | CGCAAATGGGCGGTAGGCGTG |
| Plasmids-C3aR1(R) | CTTGCCCCTTGCTCCATACCAC |

**Table S3. Docking results of C3ar1 with small molecules**

| Target | Uniprot ID | Drug | Pubchem CID | Binding energy（kcal/mol） |
| --- | --- | --- | --- | --- |
| C3ar1 | O09047 | astemizole | 2247 | -8.3 |
| C3ar1 | O09047 | terfenadine | 5405 | -8.3 |
| C3ar1 | O09047 | **papaverine** | 4680 | -8.4 |
| C3ar1 | O09047 | gossypol | 3503 | -7.3 |
| C3ar1 | O09047 | perhexiline | 4746 | -7.8 |
| C3ar1 | O09047 | **luteolin** | 5280445 | -8.4 |
| C3ar1 | O09047 | niclosamide | 4477 | -7.7 |
| C3ar1 | O09047 | digoxigenin | 15478 | -6.7 |
| C3ar1 | O09047 | helveticoside | 441860 | -6.7 |
| C3ar1 | O09047 | thiostrepton | 16154490 | -7.4 |
| C3ar1 | O09047 | medrysone | 247839 | -6.7 |
| C3ar1 | O09047 | strophanthidin | 6185 | -6.4 |
| C3ar1 | O09047 | mebendazole | 4030 | -4.7 |
| C3ar1 | O09047 | 5-azacytidine | 9444 | -5.7 |
| C3ar1 | O09047 | 3'-Azido-3'-deoxythymidine | 5726 | -7.7 |
| C3ar1 | O09047 | piperlongumine | 637858 | -6.7 |
| C3ar1 | O09047 | 3-(1-methylpyrrolidin-2-yl)pyridine | 942 | -5.9 |
| C3ar1 | O09047 | alprostadil | 5280723 | -5.3 |
| C3ar1 | O09047 | trichostatin | 444732 | -7.2 |
| C3ar1 | O09047 | **vorinostat** | 5311 | -8.7 |

The lower the Binding Energy, the better the binding effect and the higher the affinity

**Table S4. ROC information for each variable at its optimal cut-off value**

| Variable | cut-off value | Sensitivity | Specificity | Accuracy |
| --- | --- | --- | --- | --- |
| C3ar1 | 42.823 | 0.85185 | 0.82759 | 0.83529 |
| IL 6 | 18.2 | 0.92593 | 0.44828 | 0.6 |
| IL 10 | 260.78 | 0.40741 | 0.84483 | 0.70588 |
| IL 4 | 410.6 | 0.88889 | 0.63793 | 0.71765 |
| IL 1β | 39.486 | 0.66667 | 0.62069 | 0.63529 |

**Table S5. Baseline characteristics of patients with SAH**

| **Variable** | **mRS 0–2**  **(n=33)** | **mRS 3–6**  **(n=15)** | ***P* value** |
| --- | --- | --- | --- |
| **Age (years)** | 54.8 ± 8.5 | 64.7 ± 9.2 | 0.002 |
| **Sex, n (%)** |  |  | 0.755 |
| Male | 20 (60.6) | 8 (53.3) |  |
| Female | 13 (39.4) | 7 (46.7) |  |
| **BMI (kg/m²)** | 24.1 ± 3.0 | 25.0 ± 3.5 | 0.397 |
| **Hypertension, n (%)** | 10 (30.3) | 9 (60.0) | 0.064 |
| **Diabetes mellitus, n (%)** | 4 (12.1) | 3 (20.0) | 0.662 |
| **Current smoking, n (%)** | 12 (36.4) | 4 (26.7) | 0.742 |
| **Alcohol consumption, n (%)** | 10 (30.3) | 3 (20.0) | 0.727 |
| **Hunt–Hess grade≥3, n (%)** |  |  | 0.018 |
| No | 30 (90.9) | 9 (60.0) |  |
| Yes | 3 (9.1) | 6 (40.0) |  |

Abbreviations: SAH, subarachnoid hemorrhage; mRS, modified Rankin Scale; BMI, body mass index; HH, Hunt–Hess.

**Figure S1 Weighted gene co-expression network analysis (WGCNA) of the GSE36791 dataset identifies gene modules associated with SAH.**

**Figure S1 Weighted gene co-expression network analysis (WGCNA) of the GSE36791 dataset identifies gene modules associated with SAH.** **A** Hierarchical clustering dendrogram of samples and associated trait heatmap clearly distinguishing SAH patients from healthy controls (HC). **B** Analysis of soft-thresholding power shows that a power of 12 achieves optimal scale-free topology fit index and stable mean connectivity for network construction. **C** Gene dendrogram and corresponding module colors depict distinct co-expression modules identified by dynamic tree cutting. **D** Heatmap showing correlations between module eigengenes and clinical trait (SAH vs HC). **E** Functional enrichment analysis of key SAH-associated modules.

**Figure S2. Validation of C3ar1 expression levels in KO mice.**

**Figure S2. Validation of C3ar1 expression levels in KO mice. A** Representative western blot bands. **B** Density metric quantification of C3ar1 (with GAPDH as the internal reference). ^****^*P*＜0.0001.

**Figure S3. Bulk RNA-seq revealed pathways enriched for differentially expressed genes between the two groups of mice (KO vs WT).**

**Figure S3. Functional enrichment analysis of DEGs identified by bulk RNA-seq between WT and C3aR1 KO mice after SAH.**

**Figure S4. SB-505124 abolished the neuroprotective effects of C3aR1 deficiency after SAH**

**Figure S4. SB-505124 inhibits Smad2/3 phosphorylation and reverses the protective effects of C3aR1 deficiency after SAH. A** Brain water content. **B** Modified Garcia score. **C** Rotarod test. D Total time in the pole test. **E** Turn time in the pole test. **F** Representative western blot images of TGF-β1, p-Smad2/3, Smad2/3, and β-tubulin. **G–H** Quantification of TGF-β1 and p-Smad2/3 expression. Data are presented as mean ± SD (n = 6 per group). ^*^*P* < 0.05, ^**^*P* < 0.01, ^***^*P* < 0.001.

**Figure S5. The cell viability of BV2 cells treated with different concentrations of OxyHb.**

**Figure S5. The cell viability of BV2 cells treated with different concentrations of OxyHb.** ^*^*P*＜0.05, ^***^*P*＜0.001, ^****^*P*＜0.0001.

**Figure S6. C3aR1 knockdown attenuates OxyHb-induced M1 polarization in microglia**

**Figure S6. C3aR1 knockdown attenuates OxyHb-induced M1 polarization in microglia. A, B** qPCR analysis of iNOS and Arg1 mRNA expression in microglia treated with OxyHb and transfected with shRNA targeting C3aR1. **C** Representative western blot images showing protein expression levels of iNOS and Arg1 in different treatment groups. **D, E** Quantitative analysis of western blot results demonstrating relative protein expression of iNOS and Arg1 normalized to Tubulin.

**Figure S7. C3aR1 overexpression exacerbates OxyHb-induced M1 polarization in microglia**

**Figure S7. C3aR1 overexpression exacerbates OxyHb-induced M1 polarization in microglia. A, B** qPCR analysis of iNOS and Arg1 mRNA expression in microglia treated with OxyHb and transfected with plasmids targeting C3aR1. **C** Representative western blot images showing protein expression levels of iNOS and Arg1 in different treatment groups. **D, E** Quantitative analysis of western blot results demonstrating relative protein expression of iNOS and Arg1 normalized to Tubulin.

**Figure S8. Dose optimization of Vorinostat and its effects on body weight after SAH.**

**Figure S8. Effects of Vorinostat on cortical C3aR1 expression and body weight after SAH. A** ELISA analysis of cortical C3aR1 levels following treatment with different doses of Vorinostat. **B** Body weight changes in three group. Data are presented as mean ± SD (n = 6 per group). ^****^*P*＜0.0001; ns, not significant.
